# Supplementary material for: High-Throughput Bacteriophage Testing with Potency Determination: Validation of an Automated Pipetting and Phage Drop-Off Method
Source: Biomedicines. 2024 Feb 19;12(2):466. doi: 10.3390/biomedicines12020466 (PMC10886619; doi:10.3390/biomedicines12020466)
Supplement: Supplementary file 1 [file biomedicines-12-00466-s001.zip › Figure_S1.pdf]

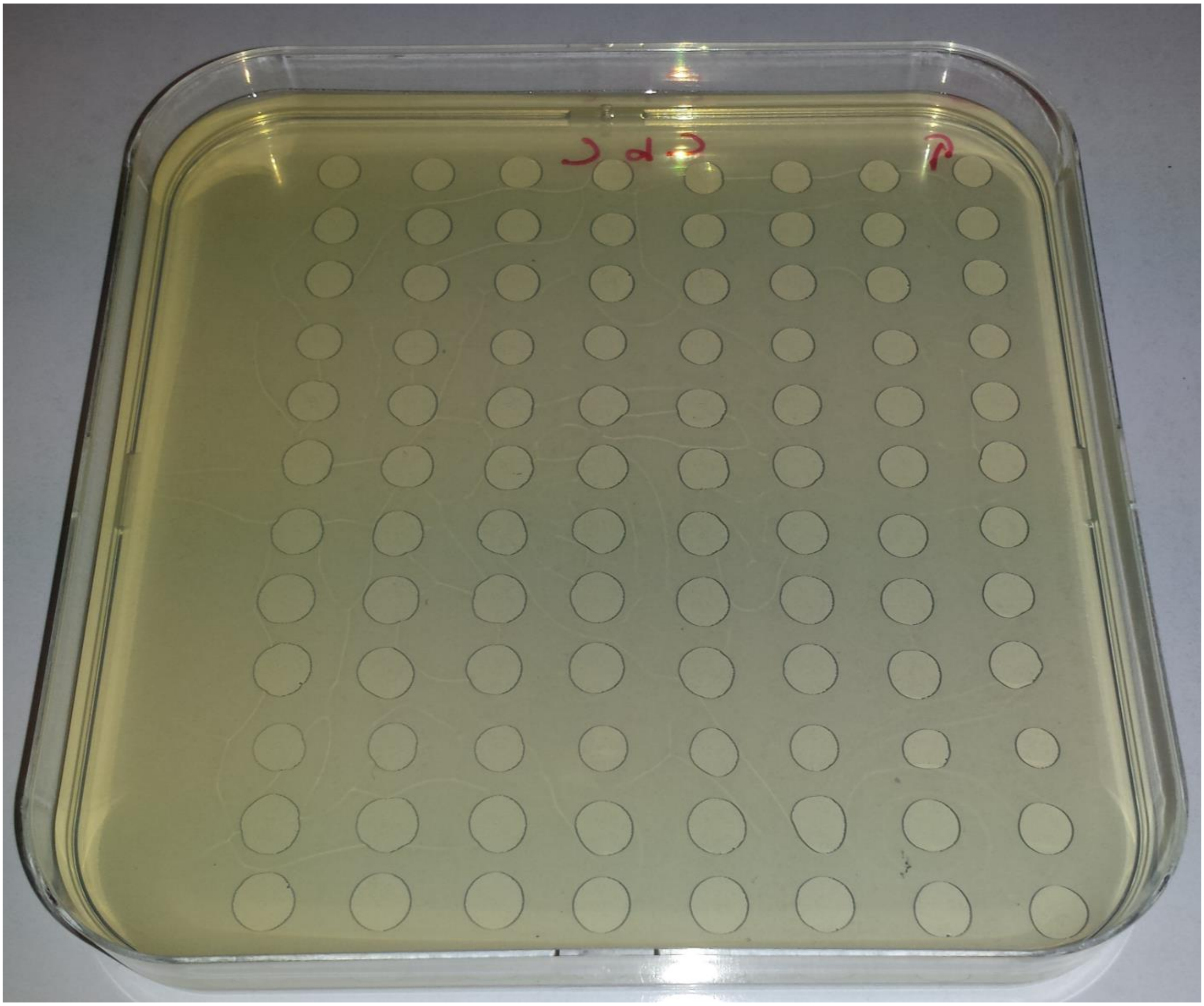

Figure S1. Representative image of an agar plate taken right after the drop-off of phage suspensions by the liquid-handling robot.
